# Alterations in the intestinal microbiome and metabolic profile of patients with cirrhosis supplemented with lactulose, *Clostridium butyricum*, and *Bifidobacterium longum infantis*: a randomized placebo-controlled trial

**DOI:** 10.3389/fmicb.2023.1169811

**Published:** 2023-04-26

**Authors:** Haifeng Lu, Xiaofei Zhu, Lingyun Wu, Xiaobin Lou, Xiaxia Pan, Bowen Liu, Hua Zhang, Lingxiao Zhu, Lanjuan Li, Zhongwen Wu

**Affiliations:** ^1^State Key Laboratory for Diagnosis and Treatment of Infectious Diseases, National Clinical Research Center for Infectious Diseases, National Medical Center for Infectious Diseases, Collaborative Innovation Center for Diagnosis and Treatment of Infectious Diseases, The First Affiliated Hospital Zhejiang University School of Medicine, Hangzhou, Zhejiang, China; ^2^Department of Infectious DiseasesHangzhou Ninth People's Hospital, Hangzhou, Zhejiang, China; ^3^Department of Radiation Oncology, The First Affiliated Hospital, Zhejiang University School of Medicine, Hangzhou, Zhejiang, China; ^4^Jinan Microecological Biomedicine Shandong Laboratory, Jinan, Shangdong, China; ^5^Research Units of Infectious Disease and Microecology, Chinese Academy of Medical Sciences, Beijing, China

**Keywords:** cirrhosis, synbiotics, shotgun metagenomics, untargeted metabolomics, *Bifidobacterium longum*

## Abstract

**Background:**

Liver cirrhosis is commonly accompanied by intestinal dysbiosis and metabolic defects. Many clinical trials have shown microbiota-targeting strategies represent promising interventions for managing cirrhosis and its complications. However, the influences of the intestinal metagenomes and metabolic profiles of patients have not been fully elucidated.

**Methods:**

We administered lactulose, *Clostridium butyricum*, and *Bifidobacterium longum infantis* as a synbiotic and used shotgun metagenomics and non-targeted metabolomics to characterize the results.

**Results:**

Patients treated with the synbiotic for 12 weeks had lower dysbiosis index (DI) scores than placebo-treated patients and patients at baseline (NIP group). We identified 48 bacterial taxa enriched in the various groups, 66 differentially expressed genes, 18 differentially expressed virulence factor genes, 10 differentially expressed carbohydrate-active enzyme genes, and 173 metabolites present at differing concentrations in the Synbiotic versus Placebo group, and the Synbiotic versus NIP group. And *Bifidobacteria* species, especially *B. longum*, showed positive associations with many differentially expressed genes in synbiotic-treated patients. Metabolites pathway enrichment analysis showed that synbiotic significantly affected purine metabolism and aminoacyl-tRNA biosynthesis. And the purine metabolism and aminoacyl-tRNA biosynthesis were no longer significant differences in the Synbiotic group versus the healthy controls group. In conclusion, although littles influence on clinical parameters in the early intervention, the synbiotic showed a potential benefit to patients by ameliorating intestinal dysbiosis and metabolic defects; and the DI of intestinal microbiota is useful for the evaluation of the effect of clinical microbiota-targeting strategies on cirrhotic patients.

**Clinical Trial Registration:**

https://www.clinicaltrials.gov, identifiers NCT05687409.

## Introduction

Liver cirrhosis is a chronic, diffuse, progressive liver disease characterized by necroinflammatory and fibrogenetic processes, which result in the structural deterioration of liver tissue, ultimately leading to liver failure (Li et al., 2021). This is always accompanied by serious complications, such as ascites, variceal bleeding, hyperuricemia, and hyperammonemia (Tonon and Piano, 2022; Wang et al., 2022). Intestinal microbiome dysbiosis is closely associated with liver cirrhosis (Milosevic et al., 2019). Mounting evidence shows that dysbiosis in patients with cirrhosis is characterized by lower abundance of bacteria that produce short-chain fatty acids and greater abundance of opportunistic pathogens (Chen et al., 2011; Qin et al., 2014; Ghosh and Jesudian, 2019). Intestinal microbiota dysbiosis is accompanied by metabolic defects in patients with liver cirrhosis that cause hepatic inflammatory injury (Frost et al., 2021; Madatali Abuwani et al., 2021).

Accumulating evidence suggests that targeting the microbiota using probiotics, prebiotics, and synbiotics may represent promising interventions for managing cirrhosis and its complications (Horvath et al., 2020). Notably, a case report showed that the reconstitution of balance in the intestinal microbiome can slow liver cirrhosis progression (Pere Ginès et al., 2021). Additionally, *Clostridium butyricum* combined with rosuvastatin ameliorates imbalances in the intestinal microbiota, liver fibrosis, and liver functional defects in patients with NAFLD (Zhu et al., 2022). Studies have also shown that *Bifidobacterium longum infantis* has health-promoting effects, such as reductions of pro-inflammatory cytokine concentration in intestinal epithelial cells (Ehrlich et al., 2020; Wang et al., 2022) and Caco-2 cells (Alvarez-Mercado et al., 2022). One study effectively applied lactulose with or without rifaximin to prevent hepatic encephalopathy (Rahimi et al., 2021), which was verified by a multi-center randomized, double blind, placebo-controlled trial (de Wit et al., 2020). However, the effects of fecal microbiota-targeting therapies in patients with liver cirrhosis have not been thoroughly investigated.

In the present study, we used synbiotic (lactulose in combination with *C. butyricum* and *B. longum infantis*, administered orally) supplementation as a microbiota-targeting strategy in patients with cirrhosis; and performed comprehensive shotgun metagenomic analysis of the intestinal microbiome and non-targeted metabolomic analysis of fasting serum. In this way, we aimed to identify how synbiotics affect microbial composition and function to influence the host metabolome, and to determine whether synbiotic supplementation benefits cirrhotic patients.

## Materials and methods

### Study design

A 12-week randomized, placebo-controlled trial was performed in accordance with the ethics guidelines of the 1975 Declaration of Helsinki and was approved by the Ethics Committee of the First Affiliated Hospital, School of Medicine, Zhejiang University (reference number: 2013–159). Adults with histologically confirmed stable cirrhosis and BMI < 25 kg m^−2^ were enrolled and assigned to receive boxes labeled A or B: one for the synbiotic, including a 10-g packet of lactulose oral solution and three capsules of probiotics (each containing >4.2 × 10^6^ CFU *C. butyricum* and > 4.2 × 10^5^ CFU *B. longum infantis*), and the other for placebo (a 10-g packet of glucose oral solution and three capsules of starch). Participants randomly chose these boxes and orally administered the contents three times daily after meals.

Participants were examined on two occasions: at baseline and 12 weeks later, and provided blood, urine, and fecal samples. We evaluated the changes in the fecal microbiome and serum metabolite concentrations between these dates. Additionally, participants were asked to keep a daily diary that included a record of their personal medication, fecal form, and any side effects of the interventions. All participants continued their antiviral therapy, and were blinded to their group allocation for the trial’s duration. Healthy volunteers were included as controls, and provided samples at baseline.

### Patients and healthy controls

Adult outpatients meeting the Chinese Medical Association (CMA) criteria for HBV-related compensated cirrhosis were recruited at the First Affiliated Hospital, School of Medicine, Zhejiang University. The inclusion and exclusion criteria are presented in Supplementary File 1. The healthy control (HC) group comprised participants matched according to the age, sex, and BMI of the LC group, who were free of chronic disease and not taking medication, including proton pump inhibitors. They were screened, enrolled, and underwent routine examination within 12 weeks. All participants provided written informed consent to participate, underwent a complete physical examination, and shared the results with the study physicians.

### Sample collection

Fecal, mid-flow urine, and fasting blood samples were collected from each participant. The fecal samples were divided into 200-mg aliquots, frozen rapidly in liquid nitrogen, and stored at −80°C until DNA extraction. Blood samples were collected in three tubes (two tubes without additives and one containing EDTA), which were delivered immediately to the clinical diagnostics laboratory for routine testing, along with the urine samples. The rest of serum samples were divided into 150-μl aliquots and stored at −80°C until Liquid chromatography-mass spectrometry (LC–MS) analysis. Participants with cirrhosis had fecal, mid-flow urine, and peripheral blood samples collected twice, once at enrollment and again after 12 weeks of taking synbiotic or placebo. The healthy controls provided their samples only once, at the initial interview.

### Fecal microbiome analysis

Fecal DNA was extracted from fecal pellets using a Qiagen DNeasy PowerSoil Kit (ref. no.12888, Germany), according to the manufacturer’s instructions. The details of DNA fragmentation and purification and sequencing library construction were same to our previous study (Lu et al., 2021), and are presented in Supplementary File 1. Sequencing was performed on an Illumina HiSeq X Ten platform (Illumina) using HiSeq X Reagent kit v2.5 (Illumina, United States), according to the manufacturer’s instructions. 150-bp paired-end reads were generated, and the raw sequencing data were submitted to the China National GeneBank DataBase (CNGBdb: https://db.cngb.org/) with the accession number CNP0003275. Details of procedures from data quality control, the quality-filtered reads co-assembly, to gene annotation and quantification were also same to our previous publication (Lu et al., 2021), and here presented in Supplementary File 1. The predicted genes were annotated using the Kyoto Encyclopedia of Genes and Genomes (KEGG) database (Aoki and Kanehisa, 2005), the carbohydrate active enzyme (CAZyme) database (Huang et al., 2018), Comprehensive Antibiotic Research Database (CARD) (Alcock et al., 2020), the Virulence Factors of Pathogenic Bacteria (VFPB) database (Liu et al., 2019), and the Gut Phage Database (GPD; http://ftp.ebi.ac.uk/pub/databases/metagenomics/genome_sets/gut_phage_database/) (Unterer et al., 2021). Dysbiosis index was used to calculate the degree of microbiota dysbiosis (see Additional file 1 for calculation details; Casén et al., 2015). Additionally, Linear Discriminant Analysis-Effect Size (LEFSe) (Zhang et al., 2022) was used to identify taxa present in differing numbers between groups. A Sankey diagram was used to visualize discriminatory microbial genes and taxa in Pavian software in R^1^ as a modular Shiny app (Breitwieser and Salzberg, 2020).

### Liquid chromatography-mass spectrometry (LC–MS)-based non-targeted metabolomic profiling

Serum samples (150 μl) were thawed at 4°C, ice-cold H_2_O (100 μl) and methanol/acetonitrile (CAN) (400 μl, 1:1, v/v) were added. The mixture was vortexed for 30 s and centrifuged for 20 min at 14,000*g* and 4°C to remove the protein. The supernatants were dried in a vacuum centrifuge and the residues were re-dissolved in 200 μl of 30% ACN (vol/vol) and transferred to insert-equipped vials (4°C) for analysis by LC–MS. Metabolites were quantitatively analyzed using a Thermo Fisher Q Exactive LC/MS system (United States) at a flow rate of 0.3 ml/min. The solvent system was A: 0.1% formic acid in water and B: 0.1% formic acid/ACN/isopropanol. The column temperature was 40°C and the injection volume was 2 μl. The details of the gradient elution and ESI source parameters are presented in Supplementary File 1. Samples were randomized to reduce the systematic error associated with instrumental drift. Quality control (QC) samples, comprising pooled serum samples from 80 participants, were injected before the first study sample, and then regularly (every eight injections) throughout the entire process, to monitor system stability and filter analytical variation. Details of the procedures from raw MS data processing, reference database-dependent ingredient definition, and calculation of metabolic concentrations were presented in Supplementary File 1. Unidentified features were excluded. For metabolites that missed in ≤25% of samples, the missing values were imputed using each metabolite’s minimum value following normalization, as previously described (Hoffman et al., 2017). The metabolite concentrations were log-transformed and Pareto-scaled within the cohort. VIP values were obtained using Orthogonal Partial Least Squares-Discriminant Analysis (OPLS-DA), which included score and permutation plot generation using the R package MetaboAnalystR (Chong and Xia, 2018).

### Statistical analysis

#### Clinical data

Statistical analysis was performed using GraphPad Prism 5 (GraphPad, San Diego, CA, United States). Data were presented as mean ± standard error of the mean (SEM). The normality of the data was tested using the Kolmogorov–Smirnov test, and normally distributed continuous data were analyzed using the two-tail paired *t*-test, whereas non-normally distributed continuous data were analyzed using the Kruskal–Wallis test. Categorical data were analyzed using the *χ*^2^ test or Fisher’s exact test.

#### Microbiome data

A gene abundance table was created for rarefaction and normalization using the MetaOMineR R package.^2^ RPKM transformations and imputation of missing values with the minimum observed values for each microbial feature were processed. A Kruskal–Wallis and *post-hoc* Dunn test were performed to analyze differences in the bacterial profile and DI among the synbiotic-treated, placebo-treated, and HC participants, and the baseline data. Differentially expressed genes were identified using the DESeq2 package with a cut-off threshold of an adjusted *p* of <0.05.

#### Metabolic data

Metabolites that discriminated between groups were identified using a VIP ≥ 1. To identify differences in individual metabolite concentrations in participants between baseline and the 12-week timepoint, the Placebo and Synbiotic, and Synbiotic and HC group concentrations were evaluated as z-scores (centered at 0 and standardized) after log2-transformation. *p* < 0.05 or a BH-adjusted *p* < 0.05 was considered to represent statistical significance. Significantly enriched KEGG pathways were identified for the differentially expressed genes using an FDR multiple testing-corrected *p* of <0.05. Significantly enriched KEGG pathways were identified for the differentially expressed genes using an FDR multiple testing-corrected *p* of <0.05. Spearman’s correlation analysis of the discriminatory microbial diversities, based on relative abundances of microbial species, and relative concentrations of metabolites was performed using R (corr.test function in the psych package). A Benjamini–Hochberg correction was applied, and MGS with at least one significant correlation at the threshold of *p* < 0.05 are represented, as previously described (Shannon et al., 2003).

Figures were created using the ggplot2 and DEseq2 packages (Xu et al., 2021).

## Results

### Participant characteristics

Eighty outpatients with cirrhosis were initially enrolled, of which 23 failed to provide fecal samples at the examination and 7 dropped out during the study for personal reasons. Therefore, 50 participants completed the study. Samples that did not pass QC were re-tested, and ultimately, samples from 29 synbiotic-treated, 21 placebo-treated, 57 pre-treated, and 30 healthy participants were analyzed. The characteristics of these participants are summarized in Table 1. No significant differences existed in the sex, age, or BMI among the groups. The serum AST and ALT activities, and TB and PLT concentrations of the participants were abnormal. However, 12 weeks of synbiotic treatment did not improve these parameters versus the placebo-treated group (all *p* > 0.05). Additionally, the MELD scores of the participants after 12 weeks did not differ from those at baseline. During the interventions, no participants experienced abdominal pain, bloating, diarrhea, or other problems.

**Table 1:** Characteristics of participants in the study.

	NIP (*N* = 57)	Synbiotics (*N* = 29)	Placebo (*N* = 21)	HCs (*N* = 30)	Value of *p*		
					NIP *vs* HCs	Synbiotics *vs* NIP	Placebo *vs* NIP
Age, Years	52.3 ± 10.6	53.6 ± 9.8	52.5 ± 10.4	48.1 ± 11.8	0.138	0.618	0.442
Sex, F/M	14/43	5/24	6/15	8/22	0.9999	0.297	0.125
BMI	22.6 ± 2.1	22.6 ± 1.5	22.2 ± 2.9	21.4 ± 2.2	0.207	0.715	0.971
ALT (U/L)	32.5 ± 22.6	36.0 ± 15.7	29.2 ± 15.3	21.2 ± 6.7	0.005	0.151	0.074
AST (U/L)	38.6 ± 17.3	35.4 ± 12.8	32.9 ± 16.4	24.3 ± 6.4	0.000	0.287	0.677
Albumin (G/L)	41.8 ± 5.2	41.0 ± 5.4	42.8 ± 4.8	45.0 ± 2.6	0.001	0.478	0.468
Globulin (G/L)	30.7 ± 6.0	32.7 ± 6.8	29.7 ± 5.8	24.0 ± 2.4	<0.001	0.266	0.166
TB (Umol/L)	28.5 ± 19.7	24.9 ± 10.7	25.3 ± 11.0	12.2 ± 4.4	<0.001	0.051	0.429
WBC	4.8 ± 7.1	3.7 ± 1.1	3.9 ± 1.1	6.0 ± 1.3	0.279	0.965	0.880
PLT	89.1 ± 51.8	87.7 ± 34.2	82.1 ± 52.7	223.9 ± 45.1	<0.001	0.846	0.735
INR	1.2 ± 0.2	1.3 ± 0.1	1.2 ± 0.1	–	–	0.408	0.514
CR	71.5 ± 12.0	69.2 ± 10.4	65.5 ± 10.9	71.4 ± 10.3	0.958	0.170	0.339
MELD	61.6 ± 3.6	61.4 ± 2.3	59.8 ± 3.5	–	–	0.148	0.795

NIP, Non-intervention patients; SYN, patients with synbiotics; PLA, patients with placebo; F, Female; M, Male; BMI, Body Mass Index; ALT, glutamic pyruvic transaminase; AST, glutamic oxaloacetic transaminase; TB, total bilirubin; WBC, White blood cell; PLT, Platelet; INR, international normalized ratio; CR, creatinine; MELD, Model for end-stage liver disease.

### Differences in the composition of the intestinal microbiota

**Figure 1:**
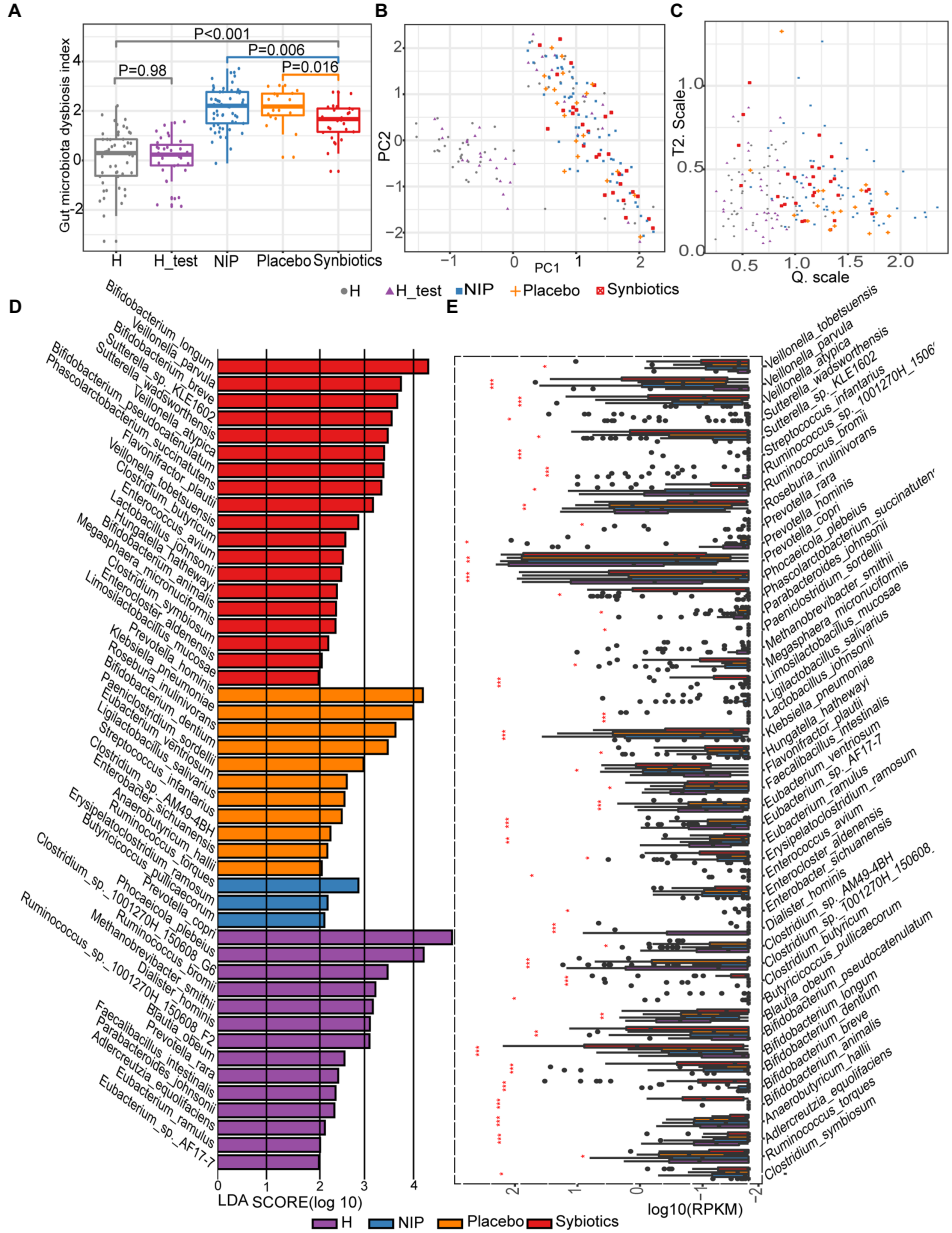
Alterations in the microbiota during synbiotic treatment. **(A)** Comparison of the DI scores of the patient and healthy control groups. **(B)** PCA for the construction of a normobiotic microbiota profile (model) to calculate the confidence ranges for the values of Hotelling’s T-square and Q statistics. **(C)** Hotelling’s T-square and Q statistics for calculating DI. *Symbol* “
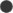
” indicates the 50 intestinal microbiota from healthy individuals in our center database, which had already been deposited in the European Bioinformatics Institute European Nucleotide Archive (ERP005860) as part of our previous study, and were age and sex-matched to the present participants (Qin et al., 2014). *Symbol* “
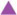
” indicates the 30 intestinal microbiotas from healthy controls in the present study for the H_test. *Symbol* “
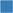
” indicates the intestinal microbiota of patients with cirrhosis at baseline. *Symbol* “
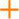
” indicates the intestinal microbiota of patients with cirrhosis after 12 weeks of placebo supplementation. *Symbol* “
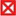
” indicates the intestinal microbiota of patients with cirrhosis after 12 weeks of synbiotic (lactulose, *Clostridium butyricum* and *Bifidobacterium longum infantis*) supplementation. **(D)** Linear discriminant analysis (LDA) effect size (LEfSe) results, based on the species that were differentially represented among the four groups. **(E)** Results of the analysis of the differentially abundant species using the Kruskal–Wallis test with the Bonferroni correction. The boxplots indicate the median and 25th to 75th percentiles. The *p*-values for each comparison are shown in Supplementary Table 1. *p < 0.05, **p < 0.01, and ***p < 0.001 for the HC group versus the NIP group, the Placebo group versus the Synbiotic group, and the Synbiotic group versus the NIP group. Red bar: Synbiotic group; yellow bar: Placebo group; blue bar: NIP group, purple bar: HC group. Further details of the comparisons are shown in Supplementary Table 1.

First, we assessed the effects of the synbiotic on the gut dysbiosis. We found that before treatment, participants with cirrhosis (NIP group) had higher DI scores than the healthy individuals (both those constituting the normobiotic reference model and those participating in the present study), and participants that underwent the 12-week synbiotic intervention showed significantly lower DIs than the NIP and Placebo groups (Kruskal–Wallis test, *p* = 0.0061 and *p* = 0.016, respectively; Figures 1A–C), but the Placebo group participants showed no change during the study.

To assess the effects of synbiotic on the composition and abundance of the intestinal microbiota, LEfSe was performed on data for the Synbiotic, Placebo, NIP, and HC groups. Of 1,329 species identified, 47 were discriminatory for particular groups, with LDA scores (log10) >2 and *p* < 0.05 (Figure 1D). The fecal microbiome of the Synbiotic group was characterized by a preponderance of *Bifidobacterium longum*, *B. breve*, *B. pseudocatenulatum*, *Veillonella parvula*, *V*. *atypica*, *Sutterella_sp_KLE1602*, *S. wadsworthensis*, *Phascolarctobacterium succinatutens*, and *Flavonifractor plautii*, while that of the Placebo group was characterized by a high abundance of *Prevotella hominis*, *Klebsiella pneumoniae*, *Roseburia inulinivorans*, *B. dentium*, *Paeniclostridium sordellii*, *Eubacterium ventriosum*, and *Ligilactobacillus salivarius* (all LDA scores (log10) >3 and *p* < 0.05; Figure. 1D). In contrast, the fecal microbiome of the HC group was dominated by *Prevotella copri*, *Phocaeicola plebeius*, *Clostridium*_sp_1001270H-150608_G6, *Ruminococcus*_sp__1001270H_150608_F2, *R. bromii*, and *Methanobrevibacter smithii* (all LDA scores (log10) >3 and *p* < 0.05; Figure 1D), and that of the NIP group was dominated by *Erysipelatoclostridium ramosum*, *R. torques*, and *Butyricicoccus pullicaecorum* (all LDA scores (log10) >2 and *p* < 0.05; Figure 1D). Additionally, the Synbiotic group was characterized by larger populations of *B. breve*, *B. longum*, *B. pseudocatenulatum*, *C. butyricum,* and *L. mucosae*; and smaller populations of *K. pneumoniae* and *Anaerobutyricum hallii* than the Placebo and NIP groups (Figure 1E; *p*-values are shown in Supplementary Table 1).

### Effects of the intervention on gene expression in the intestinal microbiota

We next determined the effect of the synbiotic on the richness and Shannon’s Diversity Index of the microbial genes using the KEGG, CAZy, ARDB, and VFDB databases. First, we assessed the alpha microbial gene diversity using the richness and Shannon metrics, and found no significant differences between the Synbiotic group and the Placebo group, and the Synbiotic group and the NIP group, except with respect to CAZyme genes in the Synbiotic group versus the Placebo group (*p* < 0.05; Supplementary Figures 1A–H). Significant differences in the richness and Shannon index of CAZyme genes were also found in the Synbiotic group versus the Placebo group (*p* < 0.05; Supplementary Figures 1B,F). However, heatmap analysis also showed differences in the abundance of discriminatory genes, including KEGG genes, CAZy genes, ARGs, and VFs, among the four groups (Supplementary Figures 1I–L; Supplementary Dataset 1).

**Figure 2:**
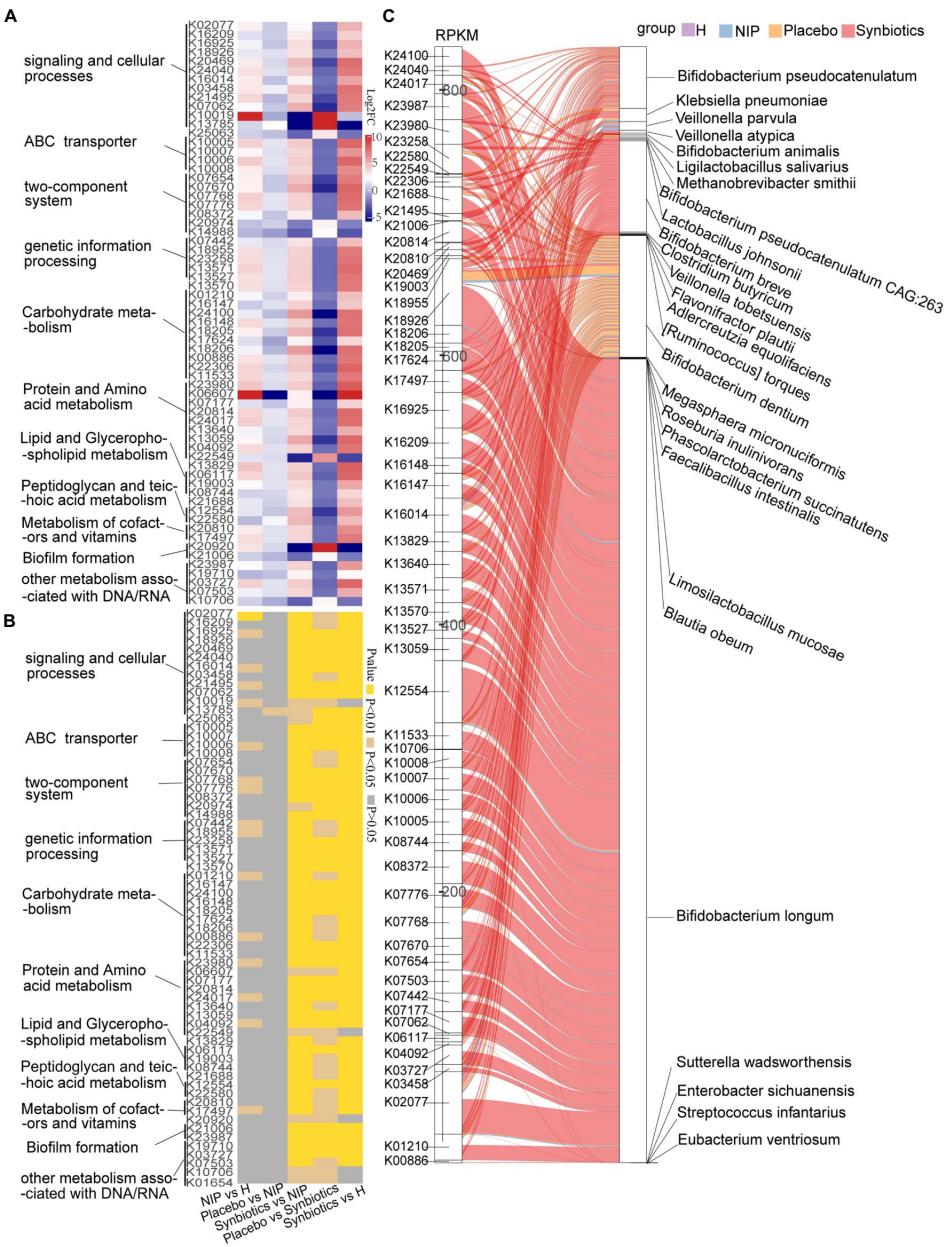
Alterations in microbial KEGG genes during the synbiotic intervention. The mean relative abundance **(A)** and associated *p-*values **(B)** for selected differentially expressed genes in the Placebo group versus the Synbiotic group and in the Synbiotic group versus the NIP group. **(C)** Correlations between differentially expressed genes and bacterial species. The boxes on the left side represent selected differentially expressed genes and the boxes on the right side represent selected discriminatory species identified using LEfSe analysis. The lines between the boxes represent the changes in each group. Red: Synbiotic group: yellow: Placebo group; blue: NIP group; and purple: HC group. Details of the comparisons in the heatmaps are shown in Supplementary Dataset 2A.

Next, discriminatory gene expression that significantly differed between the Synbiotic and Placebo groups was compared between the Synbiotic and NIP groups, the Synbiotic and HC groups, the NIP and HC groups, and the Placebo and NIP groups; and the relationships of these genes with bacterial taxa that were enriched in each group were then assessed. First, of the 382 KEGG genes that were differentially expressed between the Synbiotic and Placebo groups (*p* < 0.05; Supplementary Dataset 2A), multiple comparisons revealed that synbiotic supplementation significantly increased the expression of 56 genes involved in the following categories: signaling and cellular process, ABC transporters, two-component system, genetic information processing, transcription factors and RNA biogenesis, carbohydrate metabolism, lipid and glycerophospholipid metabolism, peptidoglycan and teichoic acid metabolism, protein and amino acid metabolism, cofactors and vitamin metabolism, and other metabolism associated with DNA/RNA (typically log_2_FC > 2; *p* < 0.05). Additionally, it reduced the expression of 10 KEGG genes (typically log_2_FC < −2; *p* < 0.05) versus the NIP group, but these genes showed no differences in expression between the Placebo and NIP groups, except with respect to TMSB10 (K13785) (Figures 2A,B). The Sankey diagram showed that *Bifidobacteria* (*B. breve*, *B. longum*, *B. animalis*, and *B. pseudocatenulatum*), *C. butyricum*, *Lactobacillus* species (*L. salivarius*, *L. johnsonii*, and *L. mucosae*), and *Veillonella* species (*V. atypica*, *V. parvula*, and *V. tobetsuensis*), which were enriched in the Synbiotic group, were responsible for the higher relative abundance of these differentially expressed genes, of which those involved in lipid pathways, amino acid and vitamin metabolism, signaling and cellular processes, and carbohydrate metabolism (Figure 2C) are of the most interest.

**Figure 3:**
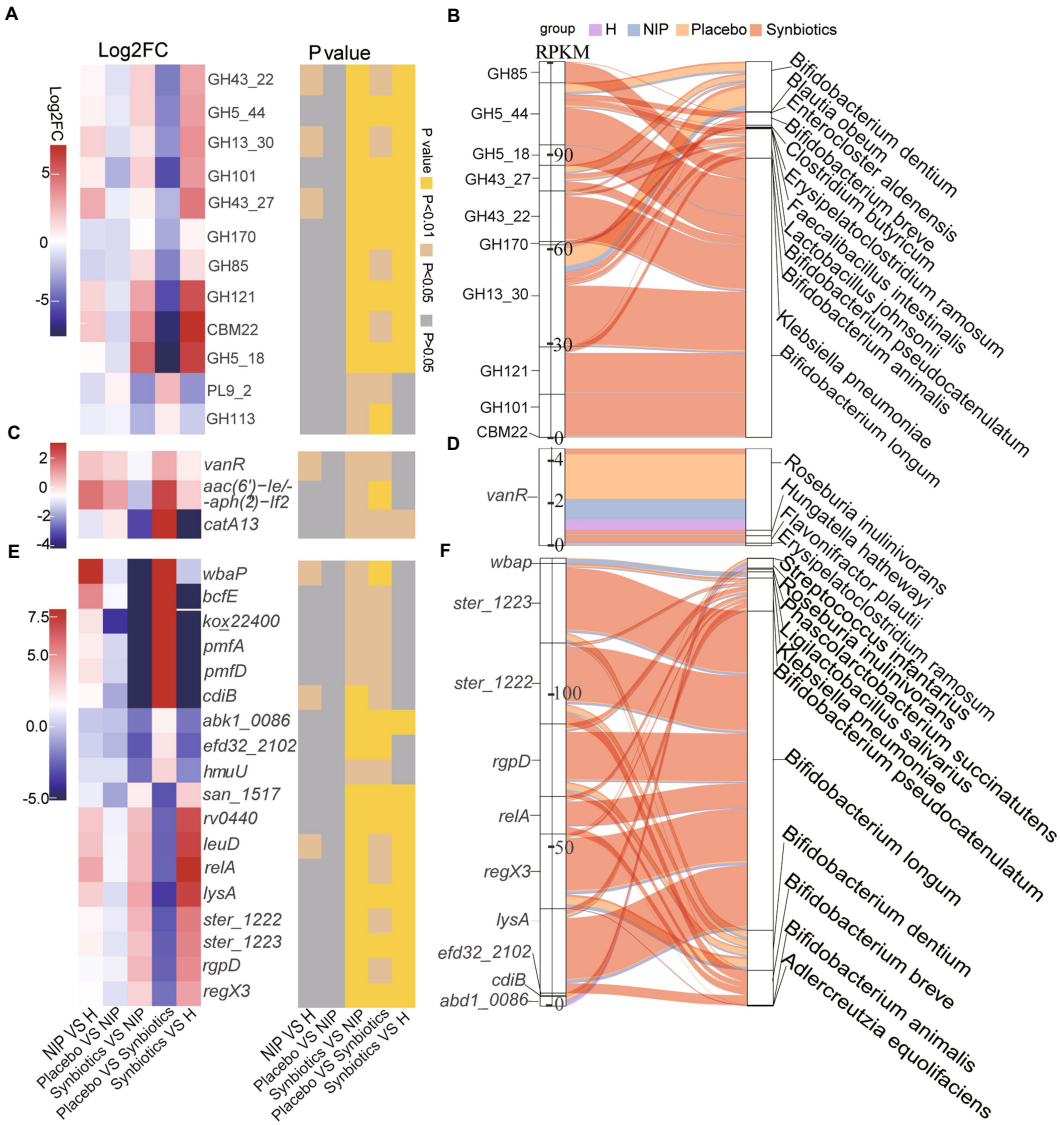
Alterations in microbial carbohydrate-active enzyme (CAZYme) genes **(A,B)**, antibiotic resistance genes (ARGs) **(C,D)** and virulence factor genes (VFGs) **(E,F)** during the synbiotic intervention. **(A,C,E)** Mean relative abundances and associated *p*-values for the selected differentially expressed genes in the Placebo group versus the Synbiotic group and in the Synbiotic group versus the NIP group. **(B,D,F)** Correlations between differentially expressed genes and bacterial species. The boxes on the left side represent the selected differentially expressed genes and the boxes on the right side represent the selected discriminatory species identified using LEfSe analysis. The lines between the boxes represent the changes in each group. Red: Synbiotic group; yellow: Placebo group; blue: NIP group; purple: HC group. Details of the comparisons in the heatmaps are shown in Supplementary Datasets 2B–D.

Second, of the 12 differentially expressed CAZyme genes in the Synbiotic versus the Placebo group (*p* < 0.05; Supplementary Dataset 2B), multiple comparisons showed that synbiotic supplementation significantly increased the expression of 10 CAZyme genes (GH43_22, GH5_44, GH13_30, GH101, GH43_27, GH170, GH85, GH121, CBM22, and GH5_18; all *p* < 0.05) and reduced that of PL9_2 and GH113 (*p* < 0.05) versus the NIP group, but these genes showed no differences in expression between the Placebo and NIP groups (Figure 3A). Additionally, the Sankey diagram showed that *Bifidobacteria* (*B. pseudocatenulatum*, *B. breve*, *B. animalis,* and *B. longum*), and especially *B. longum*, contributed to increases in the relative abundances of most of the differentially expressed CAZyme genes (Figure 3B).

Third, three differentially expressed ARGs were identified in the Synbiotic versus Placebo groups (*p* < 0.05, Supplementary Dataset 2C), the expression of which was significantly lower after 12 weeks of synbiotic supplementation (*p* < 0.05) when compared with the NIP group, but 12 weeks of placebo administration had no effect (Figure 3C). Additionally, *R. inulinivorans*, which was enriched in the Placebo group, contributed to the alteration in the relative abundance of *vanR* (Figure 3D).

Fourth, of the 67 differentially expressed VF genes in the Synbiotic versus the Placebo group (*p* < 0.05, Supplementary Dataset 2D), multiple comparisons showed that synbiotic supplementation significantly increased the expression of nine VF genes (*san*_*1517*, *rv0440*, *leuD*, *relA*, *lysA*, *ster*_*1222*, *ster*_*1223*, *rgpD*, and *regX3*; typically log_2_FC < −2, all *p* < 0.05) and reduced that of nine VF genes (*wbaP*, *bcfE*, *kox*_*22400*, *pmfA*, *pmfD*, *cdiB*, *abk1_0086*, *efd32_2102*, and *hmuU*; typically log_2_FC < −2, *p* < 0.05) versus the NIP group, but there were no differences in the expression of these genes between the Placebo and NIP groups (Figure 3E). The Sankey diagram showed that *Bifidobacteria* (*B. pseudocatenulatum*, *B. breve*, *B. animalis*, and *B. longum*), and especially *B. longum*, contributed to the higher the relative abundances of *ster*_*1222* (capsule), *ster*_*1223* (capsule), *rgpD* (capsule), *relA* ((p)ppGpp synthesis and hydrolysis), *lysA* (peptidoglycan component), and *regX3* (two-component sensory transduction protein) in the Synbiotic group (Figure 3F).

### Effects of the intervention on the intestinal phageome profile

**Figure 4:**
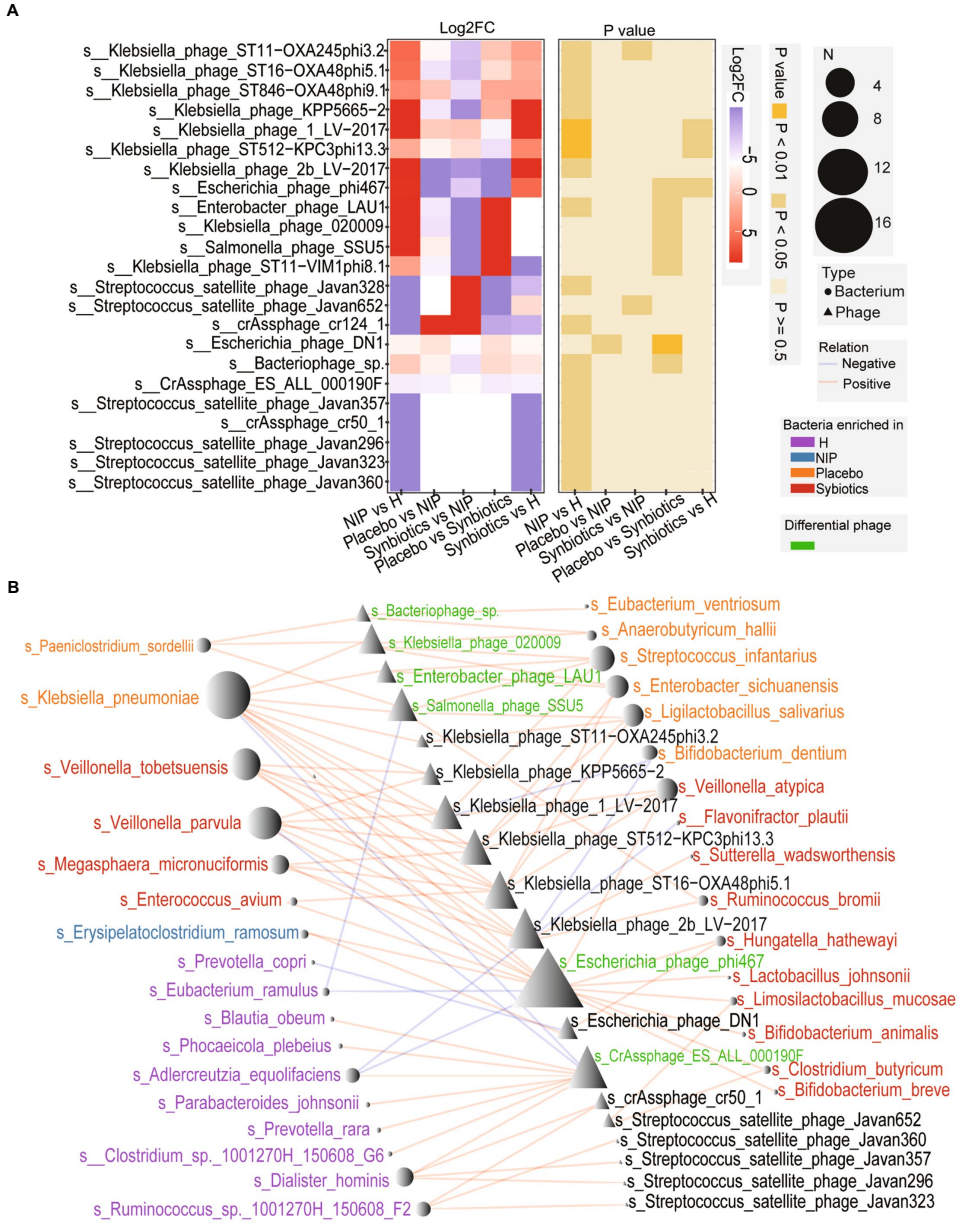
Alterations in the phageome during the synbiotic intervention. **(A)** The mean relative abundances and associated *p*-values for the selected differentially abundant phages. Further details are shown in Supplementary Table 2. **(B)** Networks connecting these phages and the selected discriminatory species identified using LEfSe analysis. Red: species enriched in the Synbiotic group; yellow: species enriched in the Placebo group; blue: species enriched in the NIP group; purple: species enriched in the HC group. Red line: positive correlation; blue line: negative correlation.

Patients with cirrhosis showed significantly greater alpha diversity of their intestinal phageome than the HC group, but no significant differences in the Shannon or richness indices, whether in the Placebo or Synbiotic groups; Supplementary Figures 2A,B). However, the total abundance of the phageome was lower in the Synbiotic group than in the Placebo or NIP groups (Supplementary Figure 2C). We performed a heatmap analysis to identify differences in low-abundance phages between the groups, and identified seven that were present in differing quantities in the Synbiotic and Placebo groups, but only two, *Klebsiella*_phage_ST11_OXA245phi3.2 and *Streptococcus*_satellite_phage_Javan 652, between the Synbiotic and NIP groups (Figure 4A; Supplementary Table 2). Among the phages which differential in the Synbiotic versus Placebo groups, differences in the abundance of four phages (s__*Salmonella*_phage_SSU5, s__*Enterobacter*_phage_LAU1, s__*Klebsiella*_phage_020009, and s__*Bacteriophage*_sp. which) were associated with the enrichment of the bacterial species in the Placebo group, and the difference in the abundance of s__*Escherichia*_phage_phi467 was associated with the enrichment of bacterial species in the Synbiotic group (Figure 4B). We also found that s__CrAssphage_ES_ALL_000190F, a member of the CrAss-like phages, was associated with the bacterial taxa that were enriched in the HC group (Figure 4B). We also performed a *Streptococcu*s_satellite-bacterium association analysis based on the relative abundance of *Streptococcus*_satellite and the bacterial species enriched in each group, and found different interactions between *Streptococcus*_satellite and fecal bacterial commensals in the Synbiotic group versus the Placebo or HC group (Supplementary Figures 2D–F).

### Effects of the intervention on the fasting serum metabolome

**Figure 5:**
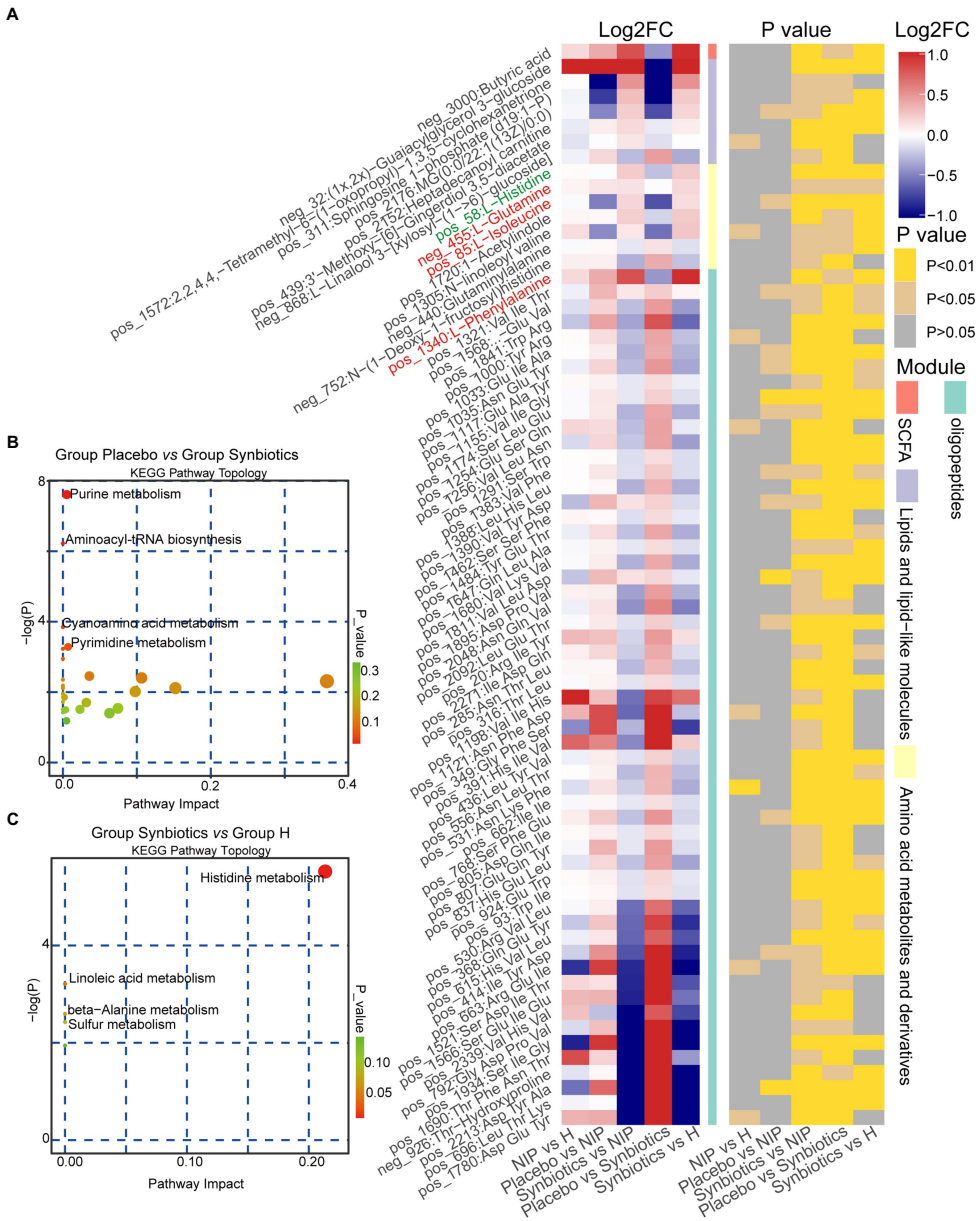
Alterations in the morning fasting serum metabolite concentrations during the synbiotic intervention. **(A)** Mean relative abundance and associated *p*-values for the selected differentially abundant metabolites in the Placebo group versus the Synbiotic group and in the Synbiotic group versus the NIP group. Metabolites in red were enriched in the aminoacyl-tRNA biosynthesis pathway, metabolites in blue were enriched in the purine metabolism pathway (including L-glutamine, in red). Details of the comparisons are shown in Supplementary Table 3 (metabolites in green were enriched in the histidine metabolism pathway). **(B,C)** Pathway enrichment analysis of the differentially abundant metabolites in the Placebo group versus the Synbiotic group **(B)** and in the Synbiotic group versus the HC group **(C)**. Each bubble represents a metabolic pathway, the position on the abscissa and the size of the bubble represents the impact of the pathway, and the position on the ordinate and the color of the bubble represent the degree of enrichment.

**Figure 6:**
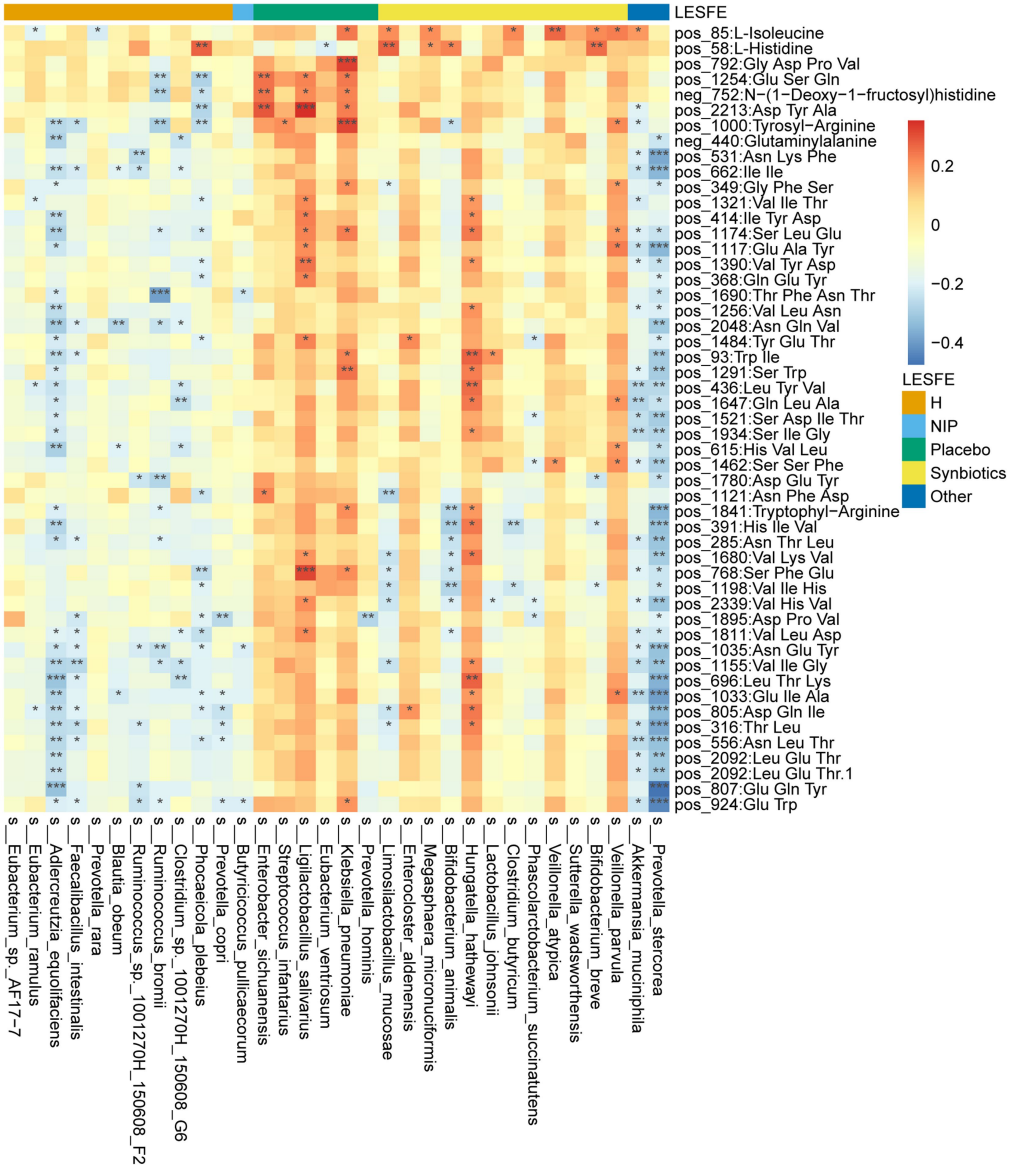
Heatmap showing the partial Spearman’s correlation coefficients for the relationships among the selected differentially abundant metabolites and the selected discriminatory species identified using LEfSe analysis. Red: positive correlations, blue: negative correlations.

We next analyzed fasting serum samples to determine whether synbiotic administration altered the metabolomes of participants by performing a non-targeted analysis of all the data acquired. We found differing concentrations of 173 metabolites in the Synbiotic versus the Placebo and NIP groups (Supplementary Table 3; *p* < 0.05). Of these, we show 71 metabolites, including 56 oligopeptides, 7 lipids and lipid-like molecules, 7 Amino acid metabolites and derivatives, and butyric acid in Figure 5A. In addition, pathway enrichment analysis based on commercial databases, including KEGG and MetaboAnalyst, showed that purine metabolism, aminoacyl-tRNA biosynthesis, cyano-amino acid metabolism, pyrimidine metabolism, and glucosinolate biosynthesis were differentially represented in the Placebo and Synbiotic groups (Figure 5B), but these five pathways did not significantly differ in their representation in the Synbiotic group versus the HC group (Figure 5C). Interestingly, *A. muciniphila*, *P. stercorea*, and six other bacterial species that were enriched in the HC group, and especially *A. equolifaciens*, showed significant negative associations with many oligopeptides, and the bacterial species enriched in the Synbiotic group showed significant positive associations with nearly half of the other differentially represented metabolites (Figure 6).

## Discussion

In the present study, we explored the relationships of differentially represented microbial genes and metabolites with the differentially enriched bacterial species in each group. First, we found that synbiotic-treated participants were characterized by high abundance of *B. longum*, *B. breve*, *B. pseudocatenulatum, C. butyrium,* and *L. mucosae*; and low abundance of *K. pneumoniae* and *A. hallii*. Second, we found that patients that underwent 12 weeks of synbiotic supplementation had lower DIs than at baseline or placebo-treated patients. Third, we identified differentially expressed KEGG genes, 18 differentially expressed VF-related genes, 10 differentially expressed CAZyme genes, and 3 differentially expressed ARGs, and 173 metabolites that were present at differing concentrations in the Synbiotic group versus the Placebo or NIP groups. Fourth, we found that *Bifidobacteria* species, and especially *B. longum*, contributed to the differential expression of these genes, and except for ARGs. Fifth, purine metabolism and aminoacyl-tRNA biosynthesis, which are associated with the hyperuricemia and hyperammonemia of cirrhosis, were significant pathways in the Placebo group versus the Synbiotic group, but not in the Synbiotic group versus the HC group. Taken together, these findings imply that a core set of metagenomic and metabolomic alterations might explain the short-term effects of microbiota-targeting strategies on the dysbiosis associated with cirrhosis, and might help determine whether patients with cirrhosis would benefit from synbiotic supplementation.

The progression of liver diseases is commonly accompanied by dysbiosis of the intestinal microbiota, including increases in the abundances of opportunistic pathogens, such as *Enterobacteriaceae*, and decreases in the abundances of health-promoting bacteria, such as *Bifidobacteria* and some butyric acid-producing bacteria (Qin et al., 2014; Ren et al., 2019). Intestinal dysbiosis is key in the pathophysiology of liver diseases and the development of complications of cirrhosis (Tilg et al., 2016; Huang et al., 2020). Emerging evidence suggests that intestinal dysbiosis probably contributes to the fibrotic/cirrhotic process and the subsequent decompensation of liver cirrhosis, and particularly to hepatic encephalopathy (Tilg et al., 2022). A substantial clinical trial conducted during recent years provided evidence of the effects of probiotics/prebiotics/synbiotics to reduce the translocation of microbial products and inflammation, resulting in hepatic injury (Sharpton et al., 2019; Vidot et al., 2019). In the present study, we did not identify significant effects of synbiotic (10 g lactulose, 1.26 × 10^7^ CFU *Clostridium butyricum*, and 1.26 × 10^6^ CFU *Bifidobacterium longum infantis*) on clinical parameters or the α-diversity of the intestinal microbiome. However, the DI index and gene heatmap analysis results showed changes in the composition and function of the intestinal microbiota, suggesting that short-term synbiotic administration ameliorates intestinal dysbiosis; increases the abundance of *Bifidobacteria*; increases the expression of CAZy genes; and increases the representation of several metabolic pathways, including carbohydrate metabolism, protein and amino acid metabolism, lipid and glycerophospholipid metabolism, ABC transporters, and signaling pathways. DI is an index that can evaluate the degree to which an individual’s microbiome deviates from that of a healthy cohort (Casén et al., 2015), and may be useful for the evaluation of clinical microbiota-targeting strategies. Here, the DIs of scores of patients with cirrhosis at baseline were higher than those of healthy individuals (including those comprising the normobiotic reference model and the HC group in the present study), which suggested that the abundances of most of the intestinal microbial species characterizing cirrhosis deviated from that characteristic of normobiosis. The DIs of patients in the Synbiotic group were significantly lower than those at baseline or those of the Placebo group. Thus, after 12 weeks of treatment, the synbiotic-treated patients showed less intestinal dysbiosis than the placebo-treated patients, which implies that synbiotics are effective at ameliorating intestinal dysbiosis in patients with cirrhosis.

Microbiota-targeting strategies that rebalance the intestinal microbiota have been beneficial for patients with cirrhosis (Liu et al., 2004; Fukui, 2017). Specifically, lactulose promotes the growth of *Bifidobacteria* and *Lactobacillus* (Bouhnik et al., 2004), and these lactate-producing bacteria promote the growth of *Veillonella* species, which generate propionic acid (Ng and Hamilton, 1971). Consistent findings were made in the present study, because *Bifidobacteria*, *Lactobacillus*, and *Veillonella* were found to be enriched in the Synbiotic group. In addition, Sankey diagram analysis showed that *B. longum*, *B. breve*, and *B. pseudocatenulatum* contribute to the identified differences in gene expression. The *B. longum* genome contains more than 60 glycosylhydrolase (GH) gene families (Li et al., 2021), and in the present study, nine differentially expressed GH family genes were identified in the Synbiotic group versus the Placebo and NIP groups that were positively associated with the bacterial species, in addition to 57 differentially expressed KEGG genes and 6 differentially expressed VF genes. This latter group comprised *relA* (encoding a GTP diphosphokinase), *regX3* (a sensory transduction protein RegX3), *ster*_*1223* (a DTDP-4-dehydrorhamnose 3,5-epimerase family protein), *ster*_*1222* (DTDP-glucose 4,6-dehydratase), *lysA* (probably encoding diaminopimelate decarboxylase), and *rgpD* (aminoacyl-tRNA synthetases), which are involved in signaling and cellular processes, and rhamnose and glucose metabolism, and we speculate that these VF genes play essential roles in the colonization and growth of *Bifidobacteria*.

We did not identify an association between *B. longum* and the differentially represented ARGs and phage genes, implying that *B. longum* is not likely to explain the differential abundances of these genes. Phages and bacteria generally undergo dynamic co-evolutionary interactions, such that alterations in each typically occur together (Jariah and Hakim, 2019). However, ecological interactions between bacteria and phages are expressed in a number of ways; for example, the presence of additional bacterial species can constrain bacteria-phage co-evolution (Blazanin and Turner, 2021). Notably, CrAssphage_ES_ALL_000190F, a CrAss-like bacteriophage that is predicted to infect Bacteroidetes hosts in the human gut microbiomes (Jonge et al., 2019), was more abundant in the metagenomes of healthy controls than in those of participants with cirrhosis, but showed no difference between the Synbiotic and Placebo groups. Of note, the networks between *Streptococcus*_satellite phages and bacteria represented in the intestinal metagenomic profiles of synbiotic-treated participants differed. Therefore, a trend toward a less abundant phageome and the interaction between bacteria and *Streptococcus*_satellite may be of interest in the study of therapeutic interventions.

The intestinal microbiota affects host metabolic homeostasis, and changes in the serum metabolite profile accompany changes in the intestinal microbiota (Kato et al., 2018). Non-targeted metabolomic profiling helps us understand how the effects of a synbiotic on the microbiota of patients with cirrhosis also affects host metabolism. We identified a number of metabolites that were present at differing concentrations, including oligopeptides, lipids and lipid-like molecules, amino acid metabolites and their derivatives, phenylglucuronide, and butyric acid. Synbiotic-treated patients showed lower fasting serum concentrations of oligopeptides and higher concentrations of lipid-like molecules than placebo-treated patients, suggesting that synbiotics may modify metabolism by increasing host protein synthesis and utilization and lipopolysaccharide (LPS) degradation. Hyperammonemia is associated with liver disease, and especially with acute or chronic liver injury (Wang et al., 2022). The aminoacyl-tRNA synthetases constitute an essential and universal family of enzymes that plays a critical role in protein synthesis (Rubio Gomez and Ibba, 2020). Patients with liver cirrhosis demonstrate a metabolic disorder that is characterized by lower protein synthesis and greater protein degradation. Therefore, liver cirrhosis is frequently accompanied by disease-related malnutrition and sarcopenia because of disease- and inflammation-induced metabolic defects (Meyer et al., 2020). Cirrhosis represents a state of anabolic resistance (Bellar et al., 2020), and nutritional supplementation is of limited value because higher serum concentrations of amino acids aggravate hyperammonemia because of the conversion of amino acids to acetyl CoA (Dasarathy, 2016). Additionally, excessive serum concentrations of uric acid, the end-product of purine metabolism in humans (Su et al., 2020), increases the risk of advanced fibrosis in patients without type 2 diabetes (Wang et al., 2022). Notably, purine metabolism and aminoacyl-tRNA biosynthesis were no longer significant differences remained in the Synbiotic group versus the HC group. We speculate that the synbiotic used in the present study has therapeutic potential, and may represent a promising means of preventing hyperuricemia and hyperammonemia in patients with cirrhosis. Additionally, the synbiotic supplementation also increased the butyric acid concentration in peripheral blood, which is considered beneficial to immune homeostasis in the other clinical trial (Cao et al., 2022).

Although these findings imply that synbiotics are beneficial for patients with cirrhosis, further large human studies and animal experiments should be conducted to determine whether and how synbiotics affect key pathways. Furthermore, linear polysaccharides (lipid-like molecules) reduce the production of inflammatory cytokines induced by LPS (Kobylkevich et al., 2021), and LPS degradation also ameliorates LPS-induced inflammation in patients. In the present study, we also found differences in the fasting serum concentrations of some polyhydroxyaldehydes and ketones (carbohydrates), organic oxygen compounds, and organoheterocyclic compounds between the groups that might be related to the differences in the expression of bacterial KEGG genes involved in carbohydrate metabolism, glycan metabolism, and cofactors and vitamin metabolism.

We acknowledge several limitations of the present study. First, this was a cross-sectional study, in which we compared the fecal microbiota and metabolite concentrations of several group, and therefore causal effects cannot be ascribed. Further experiments in animals and cell culture are necessary to elucidate the mechanistic links between the discriminatory microbes and the metabolites identified. Second, the long follow-up period influenced the compliance of the participants with respect to the intervention. We excluded several participants who did not take their assigned materials more than 10 times, which reduced the number and prolonged the length of the study period as a whole. Third, detailed information regarding the metabolic pathways or other roles of the oligopeptides identified were not available in the public dataset used; therefore, we could not draw conclusions regarding the relationships between synbiotic consumption and low fasting serum concentrations of oligopeptides in patients with cirrhosis. Despite these limitations, the results of the present study not only show effects of a synbiotic on the gut microbiota, but also emphasize the potential utility of analyzing the DI index of the microbiome to evaluate the efficacy of microbiota-targeting therapies.

## Data availability statement

The data presented in the study are deposited in the China National GeneBank DataBase (CNGBdb: https://db.cngb.org/) with the accession number CNP0003275.

## Ethics statement

The studies involving human participants were reviewed and approved by The Ethics Committee of the First Affiliated Hospital, School of Medicine, Zhejiang University (reference number: 2013-159). The patients/participants provided their written informed consent to participate in this study.

## Author contributions

ZW and LL designed the study. HL, XZ, LW, XL, BL, HZ, XP, LZ, LL, and ZW enrolled the subjects, collected clinical samples, and performed the experiment. BL, HZ, XP, LZ, and LW collected and analyzed the clinical data. HL, XZ, LW, XL, and ZW performed the omic-analysis. HL, XZ, and ZW wrote the manuscript. All authors contributed to the article and approved the submitted version.

## Funding

This work was supported by the National Natural Science Foundation of China (32170058 and 81874038), National Key Research and Development Program of China (2018YFC2000500), the major science and technology special project of Zhejiang Provincial Department of Science and Technology (2012C13018-2), Research Project of Jinan Microecological Biomedicine Shandong Laboratory (JNL-2022001A), and CAMS Innovation Fund for Medical Sciences (2019-I2M-5-045).

## Conflict of interest

The authors declare that the research was conducted in the absence of any commercial or financial relationships that could be construed as a potential conflict of interest.

## Publisher’s note

All claims expressed in this article are solely those of the authors and do not necessarily represent those of their affiliated organizations, or those of the publisher, the editors and the reviewers. Any product that may be evaluated in this article, or claim that may be made by its manufacturer, is not guaranteed or endorsed by the publisher.

## Footnotes

^1^https://www.R-project.org

^2^https://cran.r-project.org/web/packages/momr/index.html

## Glossary

ACN: Acetonitrile
ALT: Glutamic pyruvic transaminase
ARDB: Antibiotic resistance genes database
ARG: Antibiotic resistance gene
AST: Glutamic oxaloacetic transaminase
BMI: Body mass index
CARD: Comprehensive antibiotic research database
CAZyme: Carbohydrate active enzyme
CFU: Colony forming unit
CMA: Chinese medical association
CNGBdb: China National GeneBank DataBase
CoA: Coenzyme A
CR: Creatinine
DI: Dysbiosis index
DNA: Deoxyribonucleic acid
DRM: Disease-related malnutrition
dTDP: Deoxythymidine diphosphate
EDTA: Ethylenediaminetetraacetic acid
ESI: Electrospray ionization
FDR: False discovery rate
GH: Glycosylhydrolase
GPD: Gut phage database
GTP: Guanosine triphosphate
HBV: Hepatitis B virus
HC: Healthy controls
Hg: Human genome
HMDB: Human Metabolome Database
HMM: Hidden markov model
HUMAnN2: The HMP Unified Metabolic Analysis Network 2
IL: Interleukin
INR: International normalized ratio
KEGG: Kyoto encyclopedia of genes and genomes
LC/MS: Liquid chromatography/Mass spectrometer
LDA: Linear discriminant analysis
LEfSe: Linear discriminant analysis effect size
LPS: Lipopolysaccharide
MBRole: Metabolites biological role
MELD: Model for end-stage liver disease
METLIN: Metabolite mass spectral database
MGS: Metagenomic species
NAFLD: Nonalcoholic fatty liver disease
NCBI: National Center for Biotechnology information
NIP: Non-intervention patients INs Nucleotides
OPLS-DA: Orthogonal partial least squares discriminant analysis
ORF: Open reading frame
PCA: Principal component analysis
PLA: Placebo-treated patients
PLT: Platelet
QC: Quality control
RPKM: Reads per kilobase per million mapped reads
RSD: Relative standard deviation
SEM: Standard error of the mean
SYN: Synbiotics-treated patients
T2DM: Type 2 diabetes mellitus
TB: Total bilirubin
TNF: Tumor necrosis factor
tRNA: Transfer ribonucleic acid
VF: Virulent factor
VFDB: Virulence Factors Database
VIP: Variable importance in projection
VPF: Viral protein family
WBC: White blood cell
