# Supplementary material for: Alterations in the intestinal microbiome and metabolic profile of patients with cirrhosis supplemented with lactulose, Clostridium butyricum, and Bifidobacterium longum infantis: a randomized placebo-controlled trial
Source: Front Microbiol. 2023 Apr 26;14:1169811. doi: 10.3389/fmicb.2023.1169811 (PMC10170289; doi:10.3389/fmicb.2023.1169811)
Supplement: Supplementary file 6 [file Data_Sheet_3.docx]

## Title: Alterations in the intestinal microbiome and metabolic profile of patients with cirrhosis supplemented with lactulose, *Clostridium butyricum*, and *Bifidobacterium longum* *infantis*: a randomized placebo-controlled trial

## Short title: Synbiotics Effect on Cirrhosis

## Haifeng Lu^1†^，Xiaofei Zhu^2†^, Lingyun Wu^3^, Xiaobin Lou^1^, Xiaxia Pan^1^, Bowen Liu^1^, Hua Zhang^1^, Lingxiao Zhu^1^, Lanjuan Li^1,4,5*^ and Zhongwen Wu^1*^

^1^ State Key Laboratory for Diagnosis and Treatment of Infectious Diseases, National Clinical Research Center for Infectious Diseases, National Medical Center for Infectious Diseases, Collaborative Innovation Center for Diagnosis and Treatment of Infectious Diseases, The First Affiliated Hospital, Zhejiang University School of Medicine, Hangzhou, Zhejiang province, China.

^2^ Department of Infectious Diseases, Hangzhou Ninth People's Hospital, Hangzhou, Zhejiang province, China.

^3^ Department of Radiation Oncology, the First Affiliated Hospital, Zhejiang University School of Medicine, Hangzhou, Zhejiang province, China.

^4^ Jinan Microecological Biomedicine Shandong Laboratory, Jinan, Shangdong province, China.

^5^ Research Units of Infectious disease and Microecology, Chinese Academy of Medical Sciences.

**^†^** These authors have contributed equally to this work.

^*^ Correspondence: Zhongwen Wu, wuzhongwen@zju.edu.cn; Lanjuan Li, ljli@zju.edu.cn

***Methods***

***The inclusion and exclusion criteria of cirrhotic patients***

Adult outpatients meeting the Chinese Medical Association (CMA) criteria for HBV-related compensated cirrhosis were recruited at the First Affiliated Hospital, School of Medicine, Zhejiang University. The inclusion criteria were: (1) adults aged 35–60 years, (2) ultrasonographic findings confirming liver cirrhosis, (3) absence of high serum ALT and AST activities during the preceding 6 months, (4) a MELD score at the Child–Pugh A or B level (≤9), and (5) voluntary participation and provision of written informed consent. Individuals who met any of the following criteria were excluded: (1) infection with hepatitis viruses other than HBV; (2) autoimmune hepatitis, primary biliary cholangitis, primary sclerosing cholangitis, hemochromatosis, Wilson’s disease, gastrointestinal cancer, hepatocellular carcinoma, drug-induced liver injury, or severe psychiatric disorder; (3) baseline serum bilirubin concentration >3 mg/dL or platelet count <10×103 /ml; (4) excessive alcohol, cigarette, or drug use; (5) antibiotic use within the preceding 6 weeks; (6) surgery within the preceding 5 years; (7) chronic disorders associated with nutritional, metabolic, or immunological diseases; and (8) pregnancy, planned pregnancy, lactation, abdominal pain or abdominal bloating, or discontinuation of the synbiotic or placebo for >7 days.

***DNA fragmentation and purification, sequencing library construction***

The concentrations of the DNA samples obtained were quantified using a Qubit dsDNA HS Assay Kit (Invitrogen, Q32854), equalized to 20 ng/μL, and fragmented (10 μL) by sonication to 350 bp using an Ultrasonic 5-cell crusher (Covaris LE220R-plus, USA). A NEB Next® UltraTM DNA Library Prep Kit for Illumina (NEB, USA) was used to generate a DNA sequencing library, and an attribute sequence index code was added to each sample. The library quality was assessed using a Qubit® 2.0 Fluorometer (Life Technologies, Carlsbad, CA, USA) and Agilent Bioanalyzer 2100 system.

***Details of procedures of rawdata quality control*, *the quality-filtered reads co-assembly*, *gene annotation and quantification***

Raw sequencing reads were quality-controlled using KneadData (version 0.6.1, https://bitbucket.org/biobakery/kneaddata). Raw reads were processed to obtain high-quality clean reads according to the following standards: reads containing ≥10% unidentified nucleotides (Ns), containing > 50% bases with paired quality scores ≤5, belonging to the human genome, and aligned with the barcode adapter were removed. We identified host DNA by mapping with GEM(Marco-Sola, Sammeth, Guigo, & Ribeca, 2012) to the human genome (hg19) with inclusive parameters. We subsampled all samples to obtain 10 million reads. Briefly, low-quality bases were trimmed from the 3′ end of reads using Trimmomatic (version 0.36) (Bolger, Lohse, & Usadel, 2014), and the trimmed reads(Langmead & Salzberg, 2012) were compared against the ChocoPhlAn database(Danilova et al., 2019), and community-level functional metabolic pathways were identified using HUMAnN2(Song et al., 2021) against the UniRef90 protein reference database(Suzek, Huang, McGarvey, Mazumder, & Wu, 2007), with default settings. The resulting taxonomic and pathway abundance files for every sample were joined and normalized according to their relative abundances. To explore the metagenomic information for the intestinal microbiota, all the quality-filtered reads derived from the samples were co-assembled using MEGAHIT (v.1.0.3)(Li et al., 2016), and potential genes were predicted using the assembled contigs by Prokka (Seemann, 2014). Gene ≥100 bp long were retained. All the predicted genes were combined and clustered using CD HIT(Fu, Niu, Zhu, Wu, & Li, 2012) into a non-redundant gene set by setting the similarity threshold to 95%, and the relative abundances of unique genes were calculated using SALMON(Patro, Duggal, Love, Irizarry, & Kingsford, 2017).

***Details of conditions for LC-MS-based non-targeted metabolomic analysis***

Metabolites were quantitatively analyzed using a Thermo Fisher Q Exactive LC/MS system (USA) at a flow rate of 0.3 ml/min. The solvent system was A: 0.1% formic acid in water and B: 0.1% formic acid/ACN/isopropanol. The column temperature was 40°C and the injection volume was 2 μl. The linear gradient elution used was: 0.0–2.0 min A/B (90:10 v/v), 6.0–15.0 min A/B (40:60 v/v), 15.1–17.0 min water/ACN (90:10 v/v). The ESI source parameters were set as follows: spray voltage, −2.8 kV/3.0 kV; sheath gas pressure, 40 arb; auxiliary gas pressure, 10 arb; sweep gas pressure, 0 arb; capillary temperature, 320°C; and auxiliary gas heater temperature, 350°C. Samples were randomized to reduce the systematic error associated with instrumental drift. Quality control (QC) samples, comprising pooled serum samples from 80 participants, were injected before the first study sample, and then regularly (every eight injections) throughout the entire process, to monitor system stability and filter analytical variation

***Details of the procedures from raw MS data processing, reference database-dependent ingredient definition, and calculation of metabolic concentrations***

Raw MS data were acquired on the Q-Exactive using Xcalibur 4.1 (Thermo Scientific) and processed using Progenesis QI (Waters Corporation, Milford, MA, USA), according to a published protocol(Vinaixa et al., 2012). Each volatile compound's relative concentration was calculated using each volatile compound's relative area compared with the largest total peak area (100%) associated with the samples. Unidentified features were excluded. Only the present rates of those metabolite features in at least one group >80% were considered for quantification, and the relative standard deviation of those >30% in the QC samples after normalization were removed. For metabolites that missed in ≤25% of samples, the missing values were imputed using each metabolite's minimum value following normalization, as previously described(Hoffman et al., 2017). The metabolite features were assigned by comparison with chemical standards from the HMDB(Wishart et al., 2018) and METLIN(Guijas et al., 2018) databases (<http://www.hmdb.ca/> and <https://metlin.scripps.edu/>). Identified metabolites were then used as inputs into MBRole 2.0 (<http://csbg.cnb.csic.es/mbrole2/index.php>) to perform KEGG pathway enrichment analysis(Lopez-Ibanez, Pazos, & Chagoyen, 2016).

***DI analysis***

Principal component analysis (PCA) was performed to build a normobiotic microbiota profile (model). The boundary between non-dysbiotic and dysbiotic was determined by calculating confidence regions for the values of Hotelling’s T-squared and Q statistics provided by the PCA scores in the model(Casen et al., 2015). First, a normobiotic microbiota model was constructed using the relative abundances of the microbial taxa of 50 healthy intestinal microbiota, which had been deposited in the European Bioinformatics Institute European Nucleotide Archive ([ERP005860](http://www.ebi.ac.uk/ena/data/view/ERP005860)) following our previous study, and matched according to the age and sex of the participants in the present study(Qin et al., 2014). Second, the T-squared and Q statistics for a sample were calculated on the basis of the model and scaled using the confidence limits. Third, the Euclidian distances from the origin were calculated on the plot of the T-squared *vs*. Q statistics. Because the distances had a log-normal distribution, the Z score was used to identify outliers.

***Statistical analysis***

*Clinical data* Statistical analysis was performed using GraphPad Prism 5 (GraphPad, San Diego, CA, USA). Data are presented as mean ± standard error of the mean (SEM). The normality of the data was tested using the Kolmogorov–Smirnov test, and normally distributed continuous data were analyzed using the two-tail paired *t*-test, whereas non-normally distributed continuous data were analyzed using the Kruskal–Wallis test. Categorical data were analyzed using the χ^2^ test or Fisher’s exact test.

*Microbiome data* A gene abundance table was created for rarefaction and normalization using the MetaOMineR R package (<https://cran.r-project.org/web/packages/>momr/index.html). RPKM transformations and imputation of missing values with the minimum observed values for each microbial feature were processed. A Kruskal–Wallis and *post-hoc* Dunn test were performed to analyze differences in the bacterial profile and DI among the synbiotic-treated, placebo-treated, and HC participants, and the baseline data. Differentially expressed genes were identified using the DESeq2 package with a cut-off threshold of an adjusted *P* of <0.05.

***Metabolic data*** Metabolites that discriminated between groups were identified using a VIP ≥ 1. To identify differences in individual metabolite concentrations in participants between baseline and the 12-week timepoint, the Placebo and Synbiotic, and Synbiotic and HC group concentrations were evaluated as z-scores (centered at 0 and standardized) after log2-transformation. *P*<0.05 or a BH-adjusted *P*<0.05 was considered to represent statistical significance. Significantly enriched KEGG pathways were identified for the differentially expressed genes using an FDR multiple testing-corrected *P* of <0.05. Significantly enriched KEGG pathways were identified for the differentially expressed genes using an FDR multiple testing-corrected *P* of <0.05. Spearman correlation analysis of the discriminatory microbial diversities, based on relative abundances of microbial species, and relative concentrations of metabolites was performed using R (corr.test function in the psych package). A Benjamini–Hochberg correction was applied, and MGS with at least one significant correlation at the threshold of *P*<0.05 are represented, as previously described(Shannon et al., 2003).

Figures were created using the ggplot2 and DEseq2 packages(Xu et al., 2021).

**Appendix**

**Supplementary Figure 1**


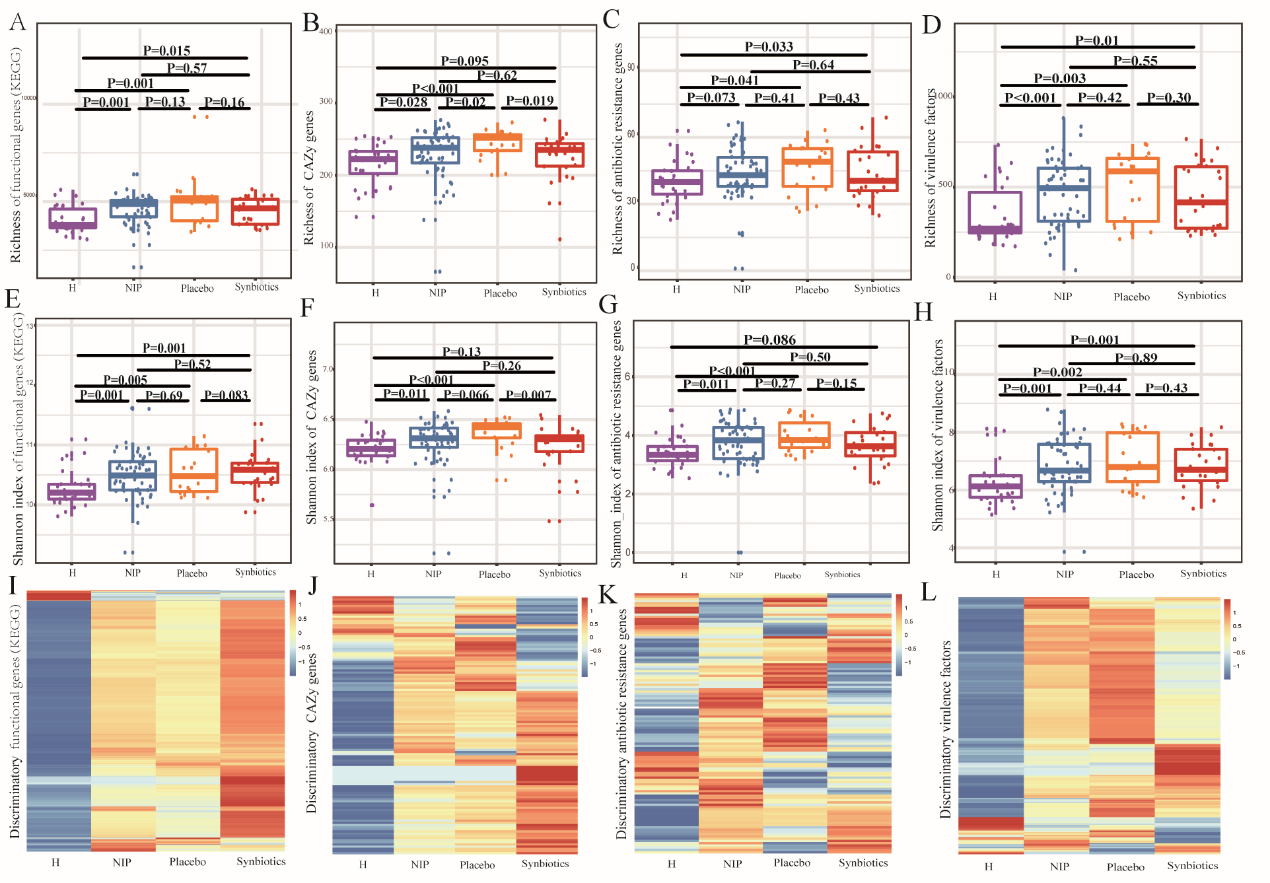


**Supplementary Figure 1** Alterations in microbial gene expression during the synbiotic intervention. **(A)**, **(B)**, **(C)**, and **(D)** Comparisons of the richness of genes (KEGG), CAZy genes, ARGs, and VF genes between the groups. **(E)**, **(F)**, **(G)**, and **(H)** Comparisons of Shannon index of genes (KEGG), CAZy genes, ARGs, and VF genes between groups. **(I)**, **(J)**, **(K)**, and **(L)** Heatmaps of the relative abundances of the differentially expressed KEGG genes, CAZy genes, ARGs, and VF genes for each group. Details of the heatmaps are shown in Supplementary Dataset 1.

**Supplementary Figure 2**


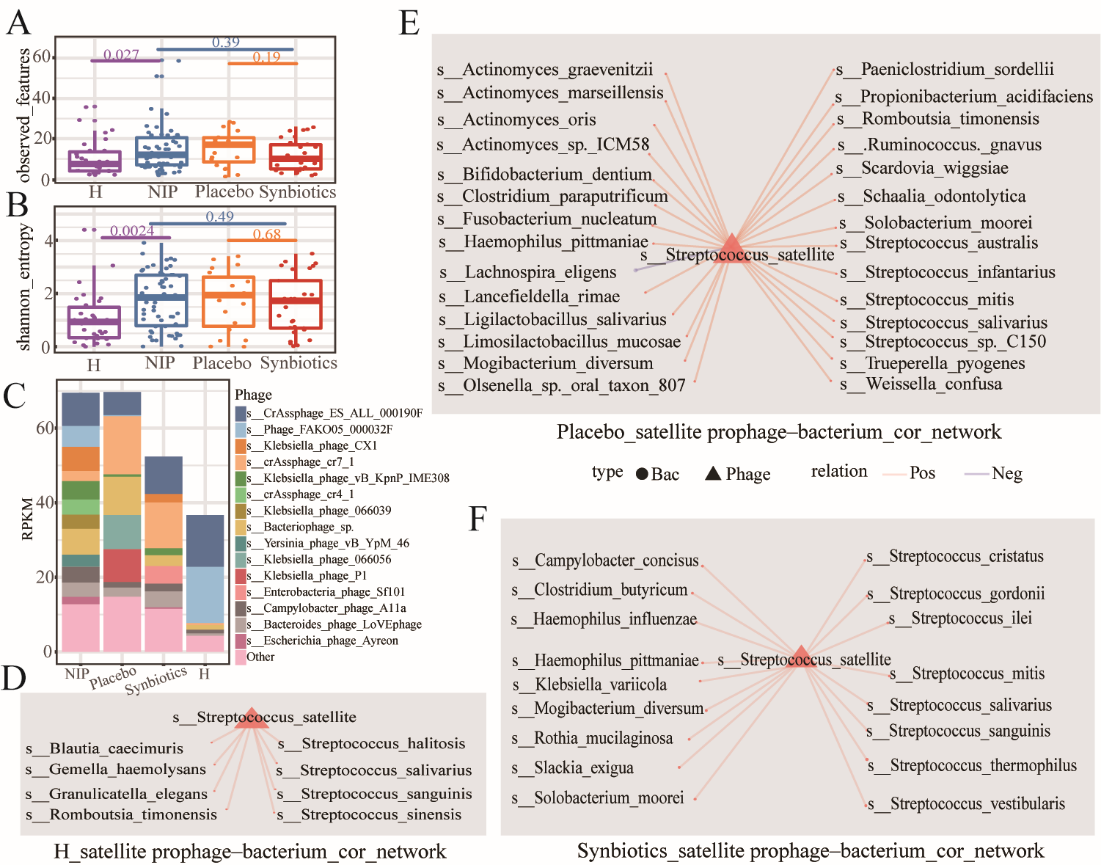


**Supplementary Figure 2** Alterations in the abundances of phages during the synbiotic intervention. **(A)** and **(B)** Comparisons of the diversity indices (a for richness, and b for Shannon index) between groups. **(C)** Mean compositions and relative abundances of the top 15 most abundant phage communities in each group at the species level. **(D)**, **(E)**, and **(F)** Network for *Streptococcus*_satellite prophage and bacterial species in the HC group (**D**), Placebo group (**E**), and Synbiotic group (**F**). Red line: positive correlation, blue line: negative correlation.

**Supplementary Table 1** Detailed data for the comparisons of the abundances of microbial taxa between groups

**Supplementary Table 2** Comparison of the phages that were differentially abundant between groups

**Supplementary Table 3** Comparison of the differentially abundant metabolites in the Placebo group *versus* the Synbiotic group and the Synbiotic group *versus* the NIP group

**Supplementary Dataset 1** Detailed data for the heatmaps shown in Supplementary Figure 1.

Supplementary Data **1A** Detailed data for the heatmap of the top 200 genes, based on the KEGG database, for each group.

Supplementary Dataset **1B** Detailed data for the heatmap of the genes derived from the CAZy database for each group.

Supplementary Data **1C** Detailed data for the heatmap of the ARGs, based on ARDB, for each group.

Supplementary Data **1D** Detailed data for the heatmap of the genes based on VFDB, for each group.

**Supplementary Dataset 2** Detailed data for the heatmaps shown in Figures 2a, 3a, 3c, and 3e.

Supplementary Dataset **2A** Comparison of the differentially expressed genes in the Placebo group *versus* the Synbiotic group and the Synbiotic group *versus* the NIP group.

Supplementary Dataset **2B** Comparison of the differentially expressed CAZy genes in the Placebo group *versus* the Synbiotic group and the Synbiotic group *versus* the NIP group.

Supplementary Dataset **2C** Comparison of the differentially expressed ARGs in the Placebo group *versus* the Synbiotic group and the Synbiotic group *versus* the NIP group.

Supplementary Dataset **2D** Comparison of the differentially expressed VF genes in the Placebo group *versus* the Synbiotic group and the Synbiotic group *versus* the NIP group.

***Reference***

Bolger, A. M., Lohse, M., & Usadel, B. (2014). Trimmomatic: a flexible trimmer for Illumina sequence data. *Bioinformatics*. 30(15), 2114-2120. doi:10.1093/bioinformatics/btu170

Casen, C., Vebo, H. C., Sekelja, M., Hegge, F. T., Karlsson, M. K., Ciemniejewska, E., et al. (2015). Deviations in human gut microbiota: a novel diagnostic test for determining dysbiosis in patients with IBS or IBD. *Aliment Pharmacol Ther*. 42(1), 71-83. doi:10.1111/apt.13236

Danilova, N. A., Abdulkhakov, S. R., Grigoryeva, T. V., Markelova, M. I., Vasilyev, I. Y., Boulygina, E. A., et al. (2019). Markers of dysbiosis in patients with ulcerative colitis and Crohn's disease. *Ter Arkh*. 91(4), 17-24. doi:10.26442/00403660.2019.04.000211

Fu, L., Niu, B., Zhu, Z., Wu, S., & Li, W. (2012). CD-HIT: accelerated for clustering the next-generation sequencing data. *Bioinformatics*. 28(23), 3150-3152. doi:10.1093/bioinformatics/bts565

Guijas, C., Montenegro-Burke, J. R., Domingo-Almenara, X., Palermo, A., Warth, B., Hermann, G., et al. (2018). METLIN: A Technology Platform for Identifying Knowns and Unknowns. *Anal Chem*. 90(5), 3156-3164. doi:10.1021/acs.analchem.7b04424

Hoffman, J. D., Parikh, I., Green, S. J., Chlipala, G., Mohney, R. P., Keaton, M., et al. (2017). Age Drives Distortion of Brain Metabolic, Vascular and Cognitive Functions, and the Gut Microbiome. *Front Aging Neurosci*. 9: 298. doi:10.3389/fnagi.2017.00298

Langmead, B., & Salzberg, S. L. (2012). Fast gapped-read alignment with Bowtie 2. *Nat Methods*. 9(4), 357-359. doi:10.1038/nmeth.1923

Li, D., Luo, R., Liu, C. M., Leung, C. M., Ting, H. F., Sadakane, K., et al. (2016). MEGAHIT v1.0: A fast and scalable metagenome assembler driven by advanced methodologies and community practices. *Methods*. 102, 3-11. doi:10.1016/j.ymeth.2016.02.020

Lopez-Ibanez, J., Pazos, F., & Chagoyen, M. (2016). MBROLE 2.0-functional enrichment of chemical compounds. *Nucleic Acids Res*. 44(W1), W201-204. doi:10.1093/nar/gkw253

Marco-Sola, S., Sammeth, M., Guigo, R., & Ribeca, P. (2012). The GEM mapper: fast, accurate and versatile alignment by filtration. *Nat Methods*. 9(12), 1185-1188. doi:10.1038/nmeth.2221

Patro, R., Duggal, G., Love, M. I., Irizarry, R. A., & Kingsford, C. (2017). Salmon provides fast and bias-aware quantification of transcript expression. *Nat Methods*. 14(4), 417-419. doi:10.1038/nmeth.4197

Qin, N., Yang, F., Li, A., Prifti, E., Chen, Y., Shao, L., et al. (2014). Alterations of the human gut microbiome in liver cirrhosis. *Nature*. 513(7516), 59-64. doi:10.1038/nature13568

Seemann, T. (2014). Prokka: rapid prokaryotic genome annotation. *Bioinformatics*. 30(14), 2068-2069. doi:10.1093/bioinformatics/btu153

Shannon, P., Markiel, A., Ozier, O., Baliga, N. S., Wang, J. T., Ramage, D., et al. (2003). Cytoscape: a software environment for integrated models of biomolecular interaction networks. *Genome Res*. 13(11), 2498-2504. doi:10.1101/gr.1239303

Song, Y., Hou, J., Kwok, J. S. L., Weng, H., Tang, M. F., Wang, M. H., et al. (2021). Whole-Genome Shotgun Sequencing for Nasopharyngeal Microbiome in Pre-school Children With Recurrent Wheezing. *Front Microbiol*. 12: 792556. doi:10.3389/fmicb.2021.792556

Suzek, B. E., Huang, H., McGarvey, P., Mazumder, R., & Wu, C. H. (2007). UniRef: comprehensive and non-redundant UniProt reference clusters. *Bioinformatics*. 23(10), 1282-1288. doi:10.1093/bioinformatics/btm098

Vinaixa, M., Samino, S., Saez, I., Duran, J., Guinovart, J. J., & Yanes, O. (2012). A Guideline to Univariate Statistical Analysis for LC/MS-Based Untargeted Metabolomics-Derived Data. *Metabolites*. 2(4), 775-795. doi:10.3390/metabo2040775

Wishart, D. S., Feunang, Y. D., Marcu, A., Guo, A. C., Liang, K., Vazquez-Fresno, R., et al. (2018). HMDB 4.0: the human metabolome database for 2018. *Nucleic Acids Res*. 46(D1), D608-D617. doi:10.1093/nar/gkx1089

Xu, Y., Zhu, J., Feng, B., Lin, F., Zhou, J., Liu, J., et al. (2021). Immunosuppressive effect of mesenchymal stem cells on lung and gut CD8(+) T cells in lipopolysaccharide-induced acute lung injury in mice. *Cell Prolif*. 54(5): e13028. doi:10.1111/cpr.13028
